# Trends and Sex Differences in Access to HIV Care with Scale Up of National HIV Treatment Guidelines in Pune, India

**DOI:** 10.1177/2325958220931735

**Published:** 2020-06-23

**Authors:** Priyanka Raichur, Sonali Pankaj Salvi, Shashikala Sangle, Amol Chavan, Smita Nimkar, Gajanan Gawande, Bharat Rewari, Jyoti Mathad, Katherine Mcintire, Amita Gupta, Ivan Marbaniang, Vidya Mave

**Affiliations:** 1 Byramjee Jeejeebhoy Government Medical College–Johns Hopkins University Clinical Research Site, Pune, Maharashtra, India; 2 Department of Medicine, BJ Government Medical College and Sassoon General Hospitals, Pune, Maharashtra, India; 3 National AIDS Control Organisation, New Delhi, New Delhi, India; 4 Department of Medicine, Center for Global Health, Weill Cornell Medicine, New York, NY, USA; 5 Department of Medicine and Infectious Diseases, Johns Hopkins University, Baltimore, MD, USA; 6 Department of Epidemiology, McGill University, Montreal, Quebec, Canada

**Keywords:** HIV/AIDS, antiretroviral therapy guidelines, sex differences, India, age at presentation

## Abstract

Test and treat is the current global standard, yet sex differences persist in access to HIV care. We assessed the differences in presentation and antiretroviral therapy (ART) uptake by sex and ART-eligibility period among ART-naive adults registered at a public ART center in India. Four ART eligibility periods were defined by programmatically determined CD4 criteria (periods I-IV: CD4 <200, <350, ≤500 cells/μL, and any CD4) between January 2005 and December 2017. Of 23 957 participants, 12 510 were male. Men consistently presented with lower median CD4 count (period I-IV, *P* < .05) and higher median age (period I-III, *P* < .001) than women. From period I to IV, median age increased in women (*P* < .0001), ART initiation time decreased in both sexes (*P* < .001), and median CD4 remained <200 cells/µL in men. Advanced HIV disease and increasing age at presentation are persistent sex-specific trends which warrant innovative HIV testing strategies in both sexes.

## Introduction

Global HIV treatment guidelines have evolved as evidence has accumulated regarding the benefits of early antiretroviral therapy (ART) initiation.^1-3^ World Health Organization (WHO) guidelines have expanded ART eligibility, raising recommended CD4 count thresholds for ART initiation and culminating in the current global standard, the universal test-and-treat approach (lifelong ART initiation for all patients testing positive for HIV, regardless of CD4 count).^4-7^ Notwithstanding the universality and nondiscriminatory practices of national HIV treatment programs across the world, studies from low- to middle-income countries reveal persistent sex disparities (male versus female) in accessing HIV care. Examples include increased risk of advanced HIV disease at presentation among men in Africa despite scale-up of HIV treatment programs and lower ART initiation among women despite cost-free ART in North America.^8-10^ Importantly, data are limited from high HIV burden countries outside sub-Saharan Africa including India, the country with the third largest global HIV burden.

Over the past 15 years, India’s national HIV care and treatment program guidelines have evolved with changing WHO recommendations. Briefly, the ART eligibility threshold was raised to CD4 count <350 cells/µL in 2011, option B+ was adopted in 2013, the ART eligibility threshold was raised to CD4 count <500 cells/µL in 2016, and universal test and treat (any CD4 count) was implemented in 2017.^11-14^ As new guidelines are implemented, it is critical to understand trends in access to HIV care.^15^ Overall, expansion of the HIV response under India’s premier governmental agency, the National AIDS Control Organization, has led to exponential growth in ART coverage from 7000 people living with HIV (PLHIV) in 2004 to over 1 million by the end of 2016.^16^ Indicators beyond ART coverage warrant analysis, including any sex-based differences.

**What Do We Already Know about This Topic?**

Despite scale-up of national HIV treatment programs, sex disparities persist in accessing HIV care.

**How Does Your Research Contribute to the Field?**

This study provides new data on the impact of expanded ART provision on sex-specific presentation to care and ART uptake under India’s national program.

**What Are Your Research’s Implications toward Theory, Practice, or Policy?**

The findings from this study highlight the need for innovative and expanded HIV testing strategies to ensure that neither men nor women are disadvantaged in timely access to ART.

Increased CD4 count at ART initiation, improved linkage to HIV care, and increased ART uptake with reduced time to ART initiation are among the expected benefits of the “test-and-treat” strategy and would reflect improved access to HIV care. Using programmatic data from a large public ART center in western India, we aimed to assess trends and sex differences in presentation to HIV care (age and CD4 count at presentation) and ART uptake (ART initiation and time to ART initiation) among ART-naive, adult patients across different phases of India’s national HIV care and treatment program. The results would inform on the real-world impact of evolving HIV guidelines with potential implications for health care policy and practice in India.

## Methods

### Study Design and Data Source

We conducted a retrospective analysis of programmatic data routinely collected among adults ≥15 years registered for HIV care at Byramjee Jeejeebhoy Government Medical College (BJGMC) and Sassoon General Hospitals (SGH) ART center in Pune, India, between January 2005 and December 2017. Exclusion criteria were no documented CD4 count at presentation and any ART initiation prior to registration. Since its establishment in 2004, over 37 000 PLHIV have registered for care at BJGMC-SGH ART center; cost-free antiretroviral drugs have been provided since 2005, and approximately 4500 patients are currently in active follow-up. Individual-level data, including sociodemographic and clinical characteristics, are routinely collected under India’s national HIV care and treatment program. Data are collected using a standard paper form and subsequently entered into an electronic database.

### Study Variables and Definitions

Study variables were abstracted from the BJGMC-SGH ART center electronic database, namely age and CD4 count at presentation to HIV care (at registration): dates of registration and ART initiation. Distinct ART eligibility phases of India’s national HIV care and treatment program were identified within the analysis period based on changing CD4 count thresholds for adults: period I (January 2005 to November 2011, CD4 <200 cells/μL), period II (November 2011 to June 2016, CD4 <350 cells/μL), period III (June 2016 to May 2017, CD4 ≤500 cells/μL), and period IV (after May 2017, any CD4 count). For each ART-eligibility period, we calculated the number of ART-eligible participants and the proportion of ART-eligible participants who initiated ART. We calculated the time to ART initiation using the registration and ART initiation dates recorded in the programmatic database.

### Statistical Analysis

Variables related to presentation to HIV care (age and CD4 count), ART eligibility, ART initiation, and time to ART initiation were summarized using descriptive statistics and compared by sex and ART eligibility period using χ^2^ and Wilcoxon rank sum tests for categorical and continuous variables, respectively; Kruskal-Wallis rank test was used to assess equality of medians in different groups. We assessed the impact of sex and age on ART initiation using univariable and age-adjusted multivariable logistic regression models. Data were analyzed using STATA version 14.2 (StataCorp 2015).

### Ethical Approval and Informed Consent

The institutional review board of Johns Hopkins University (FWA#00005752) and the ethics committee at BJGMC-SGH (FWA#00005797) approved the study. This study was a retrospective review of de-identified programmatic data collected routinely as part of the Indian national HIV program and involved less than minimal risk to the participants. As per the regulations of the Indian Council of Medical Research, which is the premier facilitator of biomedical research in India, informed consent was not required for this analysis.

## Results

### Study Population

**Figure 1.**
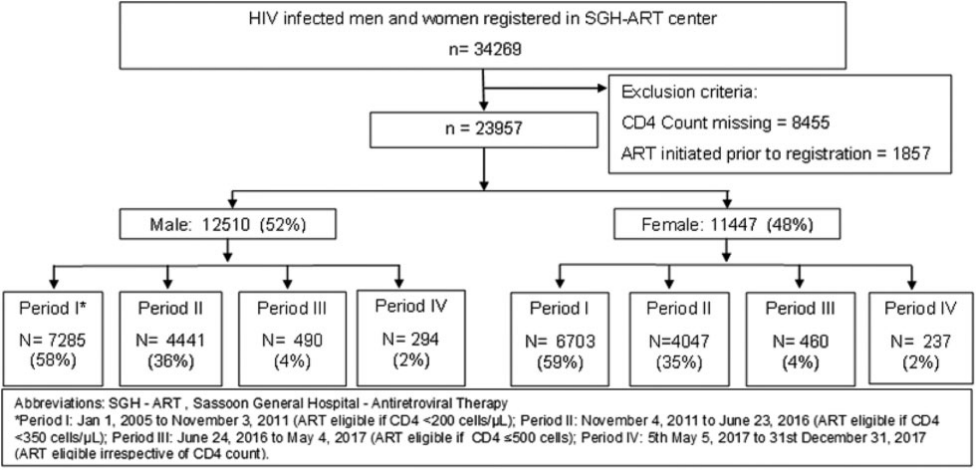
Flowchart showing study population. *Period I: January 1, 2005, to November 3, 2011 (ART eligible if CD4 <200 cells/μL), period II: November 4, 2011, to June 23, 2016 (ART eligible if CD4 <350 cells/μL), period III: June 24, 2016, to May 4, 2017 (ART eligible if CD4 ≤500 cells), and period IV: May 5, 2017, to December 31, 2017 (ART eligible irrespective of CD4 count). ART indicates antiretroviral therapy; SGH, Sassoon General Hospitals.

Of 34 269 HIV-infected adult men and women registered at BJGMC-SGH ART center during the analysis period, 23 957 (70%) fulfilled the study eligibility criteria and were included in the analysis (Figure 1). Of these, 12 510 (52%) were male, median age at presentation was 35 years (interquartile range [IQR], 30-42), and median CD4 count at presentation was 194 cells/µL (IQR, 94-364). Overall, 11 790 (80%) out of 14 777 ART-eligible participants were initiated on ART, and median time to ART initiation was 28 days (IQR, 8-122; Table 1).

**Table 1.** Trends in ART Uptake by Sex and ART Eligibility Period Among Adults Registered for HIV Care Under the National Program in Pune, India, From January 2005 to December 2017.^a^

	Overall (N = 23 957)			Period I^b^ (n =13 988)			Period II (n = 8488)			Period III (n = 950)			Period IV (n = 531)		
	Male	Female	*P*	Male	Female	*P*	Male	Female	*P*	Male	Female	*P*	Male	Female	*P*
Number (%)	12 510 (52)	11 447 (48)		7285 (52)	6703 (48)		4441 (52)	4047 (48)		490 (52)	460 (48)		294 (55)	237 (45)	
Eligible for ART at registration^c^	8148 (65)	6629 (58)	<.001	4225 (58)	3389 (51)	<.001	3192 (72)	2622 (65)	<.001	437 (89)	381 (83)	.005	294 (100)	237 (100)	
ART initiated^d^	6529 (80)	5261 (79)	.24	3256 (77)	2510 (74)	.002	2709 (85)	2231 (85)	.82	342 (78)	341 (90)	<.001	222 (76)	179 (76)	>.95
Time to ART initiation,^e^ days															
<30	3853 (59)	3013 (57)	<.001	1062 (33)	705 (28)	<.001	2294 (85)	1829 (82)	.03	298 (87)	314 (92)	.18	199 (90)	165 (92)	.47
31-90	1512 (23)	1074 (20)		1220 (37)	827 (33)		242 (9)	217 (10)		31 (10)	17 (5)		19 (9)	13 (7)	
91-180	410 (6)	305 (6)		345 (11)	249 (10)		56 (2)	51 (2)		5 (1)	4 (1)		4 (1)	1 (1)	
>180	754 (12)	869 (17)		629 (19)	729 (29)		117 (4)	134 (6)		8 (2)	6 (2)		-	-	

Abbreviations: ART, antiretroviral therapy.

^a^ All data presented as n (%) unless otherwise indicated.

^b^ Period I: January 1, 2005, to November 3, 2011 (ART eligible if CD4 <200 cells/μL), period II: November 4, 2011, to June 23, 2016 (ART eligible if CD4 <350 cells/μL), period III: June 24, 2016, to May 4, 2017 (ART eligible if CD4 ≤500 cells), and period IV: May 5, 2017, to December 31, 2017 (ART eligible irrespective of CD4 count).

^c^ According to CD4 criteria.

^d^ Of those who were ART eligible.

^e^ After ART eligibility by CD4 criteria.

### Trends and Sex Differences in Presentation to HIV Care

**Figure 2.**
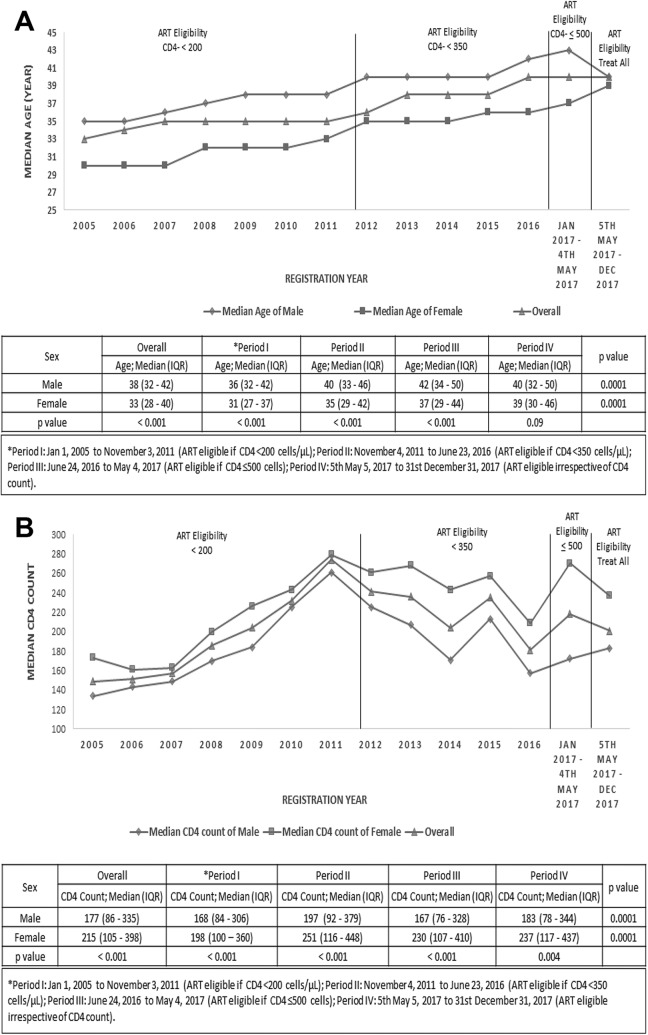
Trends in (A) median age and (B) median CD4 count at presentation by sex and ART eligibility period. A, Higher median age at presentation to HIV care among men (indicated by diamond) than women (indicated by square) during ART eligibility periods I, II, and III (*P* < .001), but median age increased significantly over the analysis period in women (*P* = .0001), with no significant sex gap in period IV (test and treat; *P* = .09). B, Men (indicated by diamond) consistently presented to HIV care with more advanced disease (as indicated by lower median CD4 count) than women (indicated by squares) across all ART eligibility periods. Median CD4 count remained below 200 cells/µL in males and below 300 cells/µL in females throughout the analysis period despite expanding ART provision. *Period I: January 1, 2005, to November 3, 2011 (ART eligible if CD4 <200 cells/μL), period II: November 4, 2011, to June 23, 2016 (ART eligible if CD4 <350 cells/μL), period III: June 24, 2016, to May 4, 2017 (ART eligible if CD4 ≤500 cells), and period IV: May 5, 2017, to December 31, 2017 (ART eligible irrespective of CD4 count).

Men tended to present for HIV care at an older age and with more advanced disease than women (Figure 2A and B). Overall, median age at presentation was 38 years (IQR, 32-45) among males versus 33 years (IQR, 28-40) among females (*P* < .001); this sex difference remained statistically significant during periods I, II, and III. However, female median age at presentation increased significantly over the analysis period from 31 years (IQR, 27-37) in period I to 39 years (IQR, 30-46) in period IV (*P* < .0001); no statistically significant sex difference was observed during period IV (Figure 2A). Overall, median CD4 count was significantly lower among males than females (177 cells/µL [IQR, 86-335] versus 215 cells/µL [IQR, 105-398]; *P* < .001). Median CD4 count at presentation increased from period I to period IV in men (168 cells/µL [IQR, 84-306] to 183 cells/µL [IQR, 78-344]; *P* < .0001) and women (198 cells/µL [IQR, 100-360] to 237 cells/µL [IQR, 117-473]; *P* < .0001), but median values remained consistently below 200 cells/µL in males and below 350 cells/µL in females across all ART eligibility periods (Figure 2B).

### Trends and Sex Differences in ART Uptake

**Figure 3.**
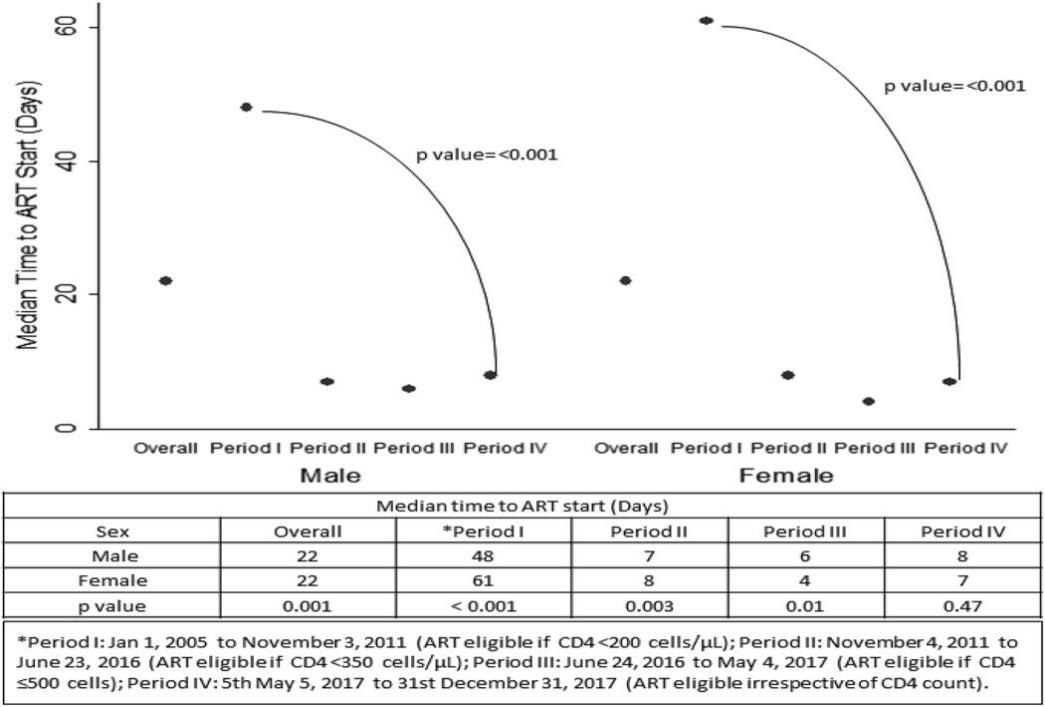
Trends in time to antiretroviral therapy (ART) initiation by sex and ART eligibility period. The median time to ART initiation (indicated by ovals) significantly decreased over the analysis period from 48 to 8 days in men (*P* < .001) and from 61 to 7 days in women (*P* < .001). No sex gap is observed in ART eligibility period IV when “test-and-treat” strategy was in effect. *Period I: January 1, 2005, to November 3, 2011 (ART eligible if CD4 <200 cells/μL), period II: November 4, 2011, to June 23, 2016 (ART eligible if CD4 <350 cells/μL), period III: June 24, 2016, to May 4, 2017 (ART eligible if CD4 ≤500 cells), and period IV: May 5, 2017, to December 31, 2017 (ART eligible irrespective of CD4 count).

Overall, more males were ART eligible than females (65% versus 58%, *P* < .001), but no significant sex difference was observed in the proportion of ART-eligible participants who initiated ART (80% versus 79%, *P* = .24; Table 1). The statistically significant sex difference in ART eligibility was observed during periods I, II, and III when CD4 count thresholds were being used and correspond to the late clinical presentation of males as described above. With expansion of ART eligibility from period I to period III, the proportion of ART-eligible participants increased from 58% to 89% in males and from 51% to 83% in females (Table 1). Equal, but low proportions of males and females (76% for both sexes) initiated ART during period IV under the test-and-treat strategy. Median time to ART initiation significantly decreased from period I to period IV in both men (48 days [IQR, 24-120] to 8 days [IQR, 3-18]; *P* < .001) and women (61 days [IQR, 27-242] to 7 days [IQR, 3-17 days]; *P* < .001), with no significant sex difference in period IV (Figure 3). The proportion of participants initiating ART within 1 month increased from period I to period IV in both men (33%-90%) and women (28%-92%; Table 1).

### Impact of Sex on ART Initiation

Univariable and age-adjusted multivariable analysis assessing the impact of sex on ART initiation showed that women had lower odds of initiating ART than men during period I (odds ratio [OR], 0.84; 95% CI, 0.76-0.94; adjusted odds ratio [aOR], 0.87; 95% CI, 0.78-0.97] but higher odds during period III (OR, 2.37; 95% CI, 1.59-3.53; aOR, 2.21; 95% CI, 1.48-3.31; Table 2).

**Table 2.** Impact of Sex on ART Uptake Using Logistic Regression Analysis.^a^

Characteristic	Period I				Period II			Period III				Period IV		
	ART eligible, n	Initiated ART, %	OR (95% CI)	aOR^b^ (95% CI)	ART eligible, n	Initiated ART, %	OR (95% CI)	ART eligible, n	Initiated ART, %	OR (95% CI)	aOR^b^ (95% CI)	ART eligible, n	Initiated ART, %	OR (95% CI)
Sex														
Male	4225	77	Ref	Ref	3192	85	Ref	437	78	Ref	Ref	294	76	Ref
Female	3389	74	**0.84 (0.76-0.94)**	**0.87 (0.78-0.97)**	2622	85	1.02 (0.88-1.18)	381	90	**2.37 (1.59-3.53)**	**2.21 (1.48-3.31)**	237	76	1.00 (0.67-1.49)
Age, years														
<40	5272	75	Ref		3007	85	Ref	381	88	Ref		262	74	Ref
>40	2342	78	**1.17 (1.04-1.31)**		2807	85	0.98 (0.85-1.14)	437	80	**0.56 (0.38-0.82)**		269	77	1.12 (0.76-1.67)

Abbreviations: aOR, adjusted odds ratio; ART, antiretroviral therapy; OR, odds ratio.

^a^ Defined as: period I (January 1, 2005, to November 3, 2011: ART eligible if CD4 <200 cells/μL), period II (November 4, 2011, to June 23, 2016: ART eligible if CD4 <350 cells/μL), period III (June 24, 2016, to May 4, 2017: ART eligible if CD4 ≤500 cells/μL), and period IV (May 5, 2017, to December 31, 2017: ART eligible irrespective of CD4 count).

^b^ Adjusted for age. The values in bold indicate statistically significant findings with *P*-value at 0.05 alpha level.

## Discussion

This retrospective study aimed to assess trends and sex differences in access to HIV care across evolving national guidelines in India from 2005 to 2017. Our analysis of programmatic data from a large, public ART center identifies promising trends regarding presentation to HIV care and ART uptake, including increasing CD4 counts at presentation and decreasing time to ART initiation, as well as diminished sex disparities in ART uptake, but also highlights areas of concern, namely persistently suboptimal ART initiation and presenting CD4 counts even under the test-and-treat strategy. Overall, this study provides new data informing on the impact of expanded ART provision under India’s national program. Our findings highlight that additional efforts are needed beyond the scope of current strategies to optimize early HIV diagnosis and effective linkage to HIV care and that barriers to ART initiation warrant further investigation and intervention.

We observed a trend of increasing CD4 count at presentation in our study among both sexes, an expected benefit of the universal test-and-treat strategy. However, median CD4 counts remained persistently low despite expansion of ART provision, especially among men who consistently presented with more advanced HIV disease than women. Similar trends have been reported in other settings.^8,17^ Late presentation to HIV care is associated with increased morbidity, mortality, and increased risk of HIV transmission, which could potentially reduce or even negate advantages of the test-and-treat approach.^18^ Importantly, gender expectations impact behavior in accessing HIV services, including testing and care. Fear of stigma and discrimination within the family and community, intimate partner violence, caregiver responsibilities, and a lack of decision-making power act as barriers for women in seeking timely HIV care. Concurrently, traditional male ideologies where men perceive themselves as tougher and capable of withstanding minor illnesses could play a role in men.^19-21^ Studies investigating gender-specific barriers to accessing HIV testing and effectiveness of linkage to HIV care are needed in Indian settings, and potential interventions should address these differences.

An interesting finding among women was the trend of increasing age at presentation to HIV care. Notably, a study in rural South Africa found that partner age disparity with “late” marriage among men in the region compounded by older men remaining alive and in active sexual networks for longer time periods contributed to rising HIV incidence among older women.^22^ The dynamics, however, are quite different in India where the average age of marriage remains low (17-24 years).^23^ Heterosexual contact is the most common mode of HIV transmission among adults in India, and excluding high risk groups, contact with a spouse who engages in high-risk behavior is the most common source of infection among Indian women.^24^ Although the prevention of parent-to-child HIV transmission (PPTCT) program provides effective screening among pregnant women, married monogamous nonpregnant women remain a vulnerable group in need of targeted interventions to improve HIV testing coverage and linkage to care as well as partner HIV disclosure.^25^

Improved ART uptake is another goal expected to accompany scale-up of HIV treatment guidelines. In our setting, time to ART initiation decreased dramatically when HIV treatment guidelines were expanded with the vast majority of men and women initiating ART within 30 days of presentation to care. Improvement in ART initiation patterns in women may indicate success of programmatic interventions focused on women.^14^ Notably, during phases of the national program when ART eligibility was based on CD4 criteria, we found that a higher proportion of men initiated ART in earlier phases and a higher proportion of women initiated ART in later years, yet no sex difference was observed under test and treat. These trends highlight the advantage of the more inclusive test-and-treat approach to ensure that neither men nor women are disadvantaged in starting ART. However, overall, suboptimal proportions of males and females initiated ART even under the test-and-treat strategy. To achieve the Joint United Nations Programme on HIV and AIDS target of ART for 90% of diagnosed PLHIV, studies are needed to explore reasons for deferring ART initiation so that barriers, including any sex-specific barriers, may be addressed using informed interventions.

Our study has limitations. Although based on data from a large HIV treatment program, this analysis was limited by incomplete information in the ART center electronic database. Adults with no recorded CD4 count at registration were necessarily excluded (n = 8455). In addition, the proportion of ART-eligible participants initiated on ART could have been underestimated; information was not available concerning those who registered for HIV care and were ART eligible but transferred out to another center and started treatment elsewhere. Despite these limitations, this analysis provides valuable insights regarding the impact of expanding HIV treatment guidelines on access to HIV care in India, identifying specific areas warranting further study and intervention toward optimization of HIV care.

In conclusion, sex disparities in ART uptake appear to have diminished with scale-up of HIV treatment guidelines under India’s national HIV care and treatment program. However, ART initiation remains suboptimal overall, and sex-specific trends in presentation to HIV care persist even under the test-and-treat strategy. The trends that men present with particularly advanced HIV disease and women present at older ages highlight the need to expand both HIV testing services and strategies for effective linkage to care beyond current recommendations. Approaches warranting exploration include the WHO recommended provider-initiated testing and counseling strategy to improve testing in men, targeted testing in “ever married” women, and integration of PPTCT services with existing health programs to increase the coverage of HIV screening.^26,27^ Further, studies in Indian settings are needed to explore factors underlying late age at presentation in women and barriers to ART initiation among men and women.
